# Supplementary figures and images for: Prediction modelling studies for medical usage rates in mass gatherings: A systematic review
Source: PLoS One. 2020 Jun 23;15(6):e0234977. doi: 10.1371/journal.pone.0234977 (PMC7310685; doi:10.1371/journal.pone.0234977)

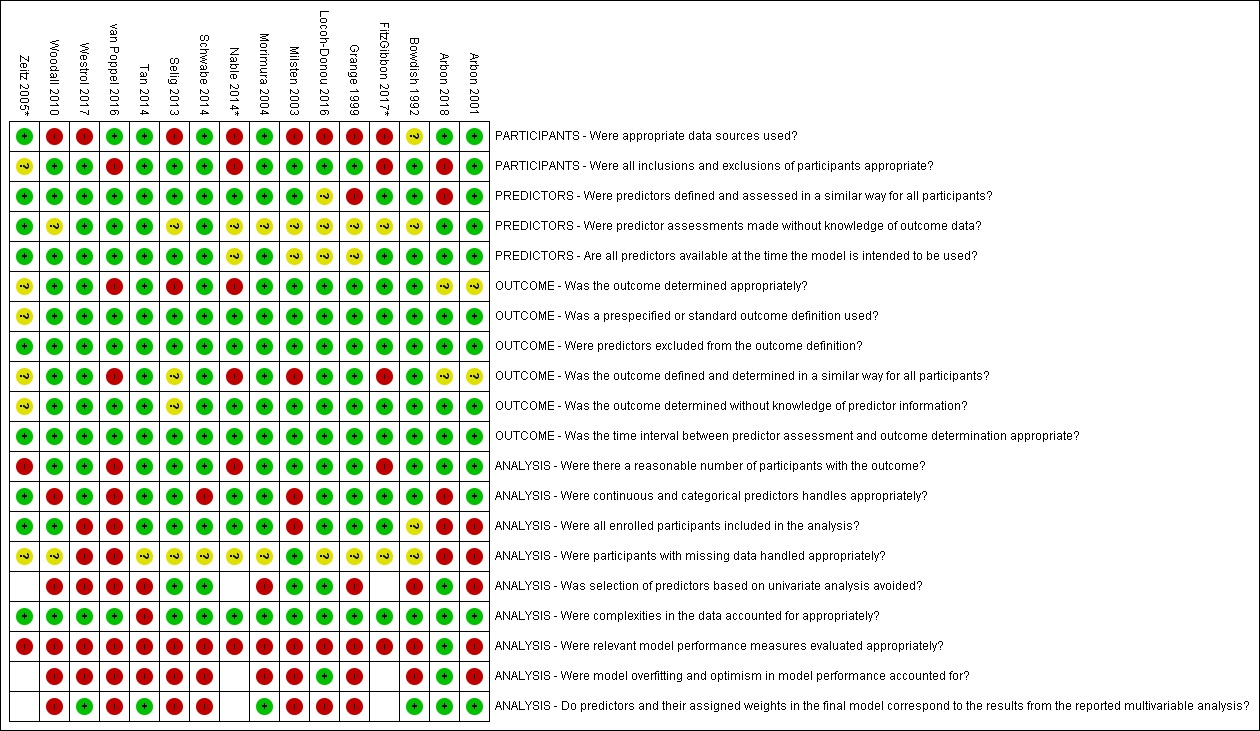

Supplement: S1 Fig — Low risk of bias (answers ‘yes’ or ‘probably yes’ to signalling questions), high risk of bias (answers ‘no’ or ‘probably no’ to signalling questions), unclear (answer ‘no information’ to signalling questions). *Studies that applied observations from few mass gatherings to another prediction model (FitzGibbon 2017, Nable 2014, Zeitz 2005): items not applicable. (TIF) [file pone.0234977.s001.tif]

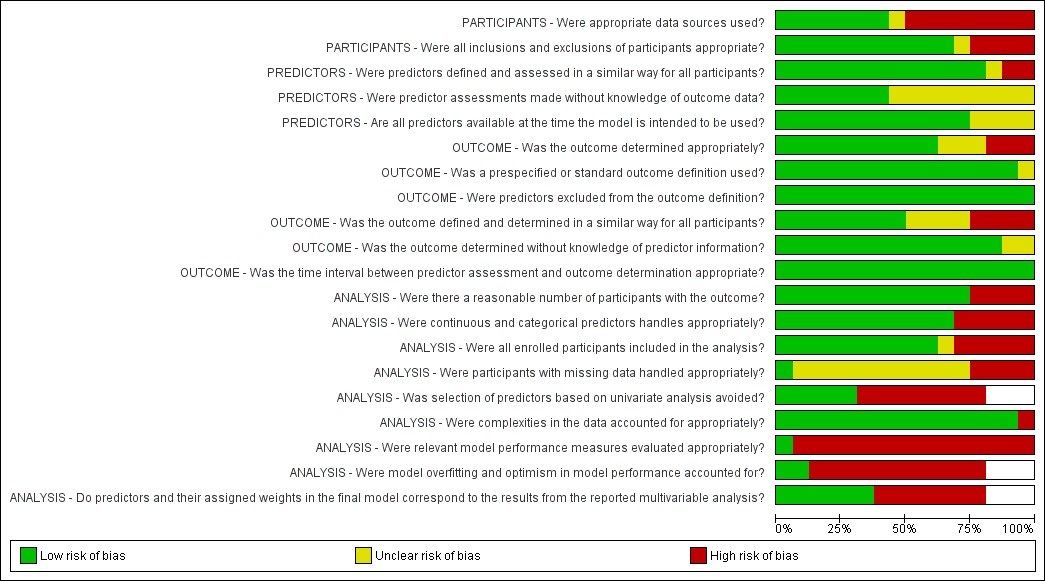

Supplement: S2 Fig — (TIF) [file pone.0234977.s002.tif]

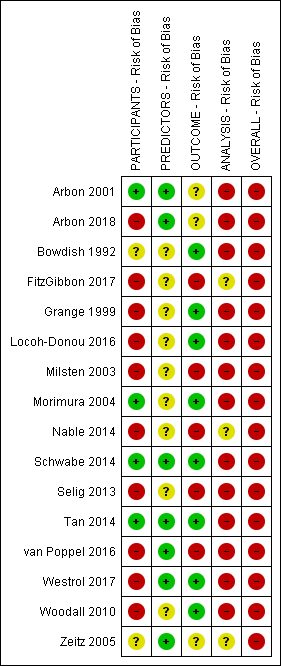

Supplement: S3 Fig — Low risk of bias, high risk of bias, unclear. (TIF) [file pone.0234977.s003.tif]

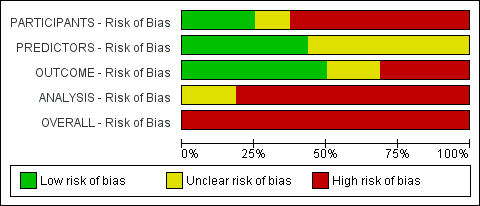

Supplement: S4 Fig — (TIF) [file pone.0234977.s004.tif]

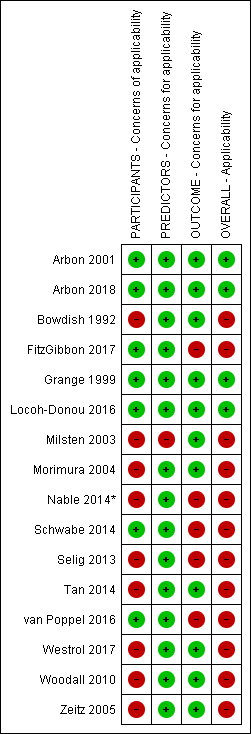

Supplement: S5 Fig — Low risk of bias, high risk of bias, unclear. (TIF) [file pone.0234977.s005.tif]

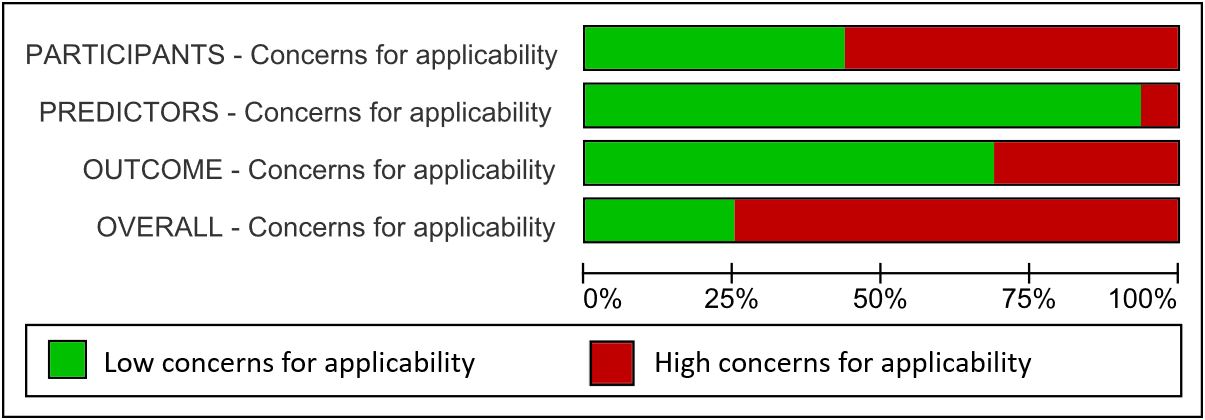

Supplement: S6 Fig — (TIF) [file pone.0234977.s006.tif]
