# Supplementary material for: Prediction modelling studies for medical usage rates in mass gatherings: A systematic review
Source: PLoS One. 2020 Jun 23;15(6):e0234977. doi: 10.1371/journal.pone.0234977 (PMC7310685; doi:10.1371/journal.pone.0234977)
Supplement: S1 Table — r: correlation coefficient; RR: risk ratio; MW U: Mann-Whitney U. £ No raw data/SD’s available (or specify), effect size and CI cannot be calculated; ¥ Imprecision (large variability of results); † Imprecision (lack of data). (DOCX) [file pone.0234977.s007.docx]

| **Author, year, Country** | **Outcome** | **Predictor** | **Effect size** | **Full model equation available?** |
| --- | --- | --- | --- | --- |
| Arbon, 2001, Australia | Total number of patient presen­tations (TOTNUM) | Multivariable model including:  seated vs. mobile;  bounded vs. unbounded;  indoor vs. outdoor;  outdoor vs. indoor;  sporting manifestations vs. non-sporting manifestations;  humidity (%);  attendance;  day + night vs. only day or night | Statistically significant with R²=0.64:  TOTNUM = -78.2 - 31.5*seated + 84.6*bounded + 42.4*indoor + 81.3*outdoor – 20.4*sport – 0.62*humidity – 0.00046*attendance + 0.000016*humidity*attendance + 20.1*daynight  *With higher expected TOTNUM for mobile, bounded, outdoor, nonsporting, day+night manifestations, and for decreasing humidity and increasing attendance* | Yes |
| Arbon, 2018, Australia | Total number of presentations | Regression tree including:  type of manifestations; attendance; duration; humidity; temperature | Regression tree: see Appendix B in the paper by Arbon et al. (2018) | Yes (cfr. Regression tree) |
| Bowdish, 1992, USA | Total number of patient presen­tations (N) | Multivariable model:  dew point  temperature  humidity  % sunshine  wind speed  excitement factor | Statistically significant:  N = -109.35 + 4.62*dew point (r=0.91, p<0.01)  *With higher N for increasing dew point*  Not statistically significant:  r=0.75, p=0.05†£  Not statistically significant†£  Not statistically significant†£  Not statistically significant†£  Not statistically significant†£ | Yes |
| Grange, 1999, USA | PPR (N/10,000) | music type  attendance  temperature  location | Statistically significant with pseudo-R²=0.04  RR=2.5, 95%CI [2.0-3.0] (p<0.0001*)  *With higher PPR in rock concerts vs. non-rock concerts*  not statistically significant:  Spearman rho=0.046 †£ (p=0.356*)  not statistically significant:  Spearman rho=-0.058 †£ (p=0.247)  not statistically significant:  MW U †£ (p=0.325) | No |
| Locoh-Donou, 2016, USA | PPR (N/10,000) | Multivariable model (adjusted for alcohol, heat index, % seating):  outside vs inside  unbounded vs bounded  no free water vs free water  no climate control vs climate control  type of manifestation: athletic manifestations vs football  type of manifestation: concerts vs football  type of manifestation:  public exhibitions vs football | Statistically significant:  RR = 2.705, 95%CI [1.586-4.614] (p<0.001)  *With higher PPR for outside manifestations*  Statistically significant:  RR = 4.627, 95%CI [1.784-12.005] (p=0.002)  *With higher PPR for unbounded manifestations*  Statistically significant:  RR = 2.011, 95%CI [1.290-3.133] (p=0.002)  *With higher PPR for no free water*  Statistically significant:  RR = 2.784, 95%CI [1.571-4.936] (p<0.001)  *With higher PPR for no climate control*  Not statistically significant:  RR = 0.479, 95%CI [0.229-1.002] (p=0.051) ¥  Not statistically significant:  RR = 0.536, 95%CI [0.284-1.009] (p=0.053) ¥  Not statistically significant:  RR = 0.826, 95%CI [0.471-1.449] (p=0.506) ¥ | No |
| Milsten, 2003, USA | PPR (N/10,000) | Multivariable model:  type of manifestation: football vs baseball  type of manifestation: rock concerts vs baseball  heat index: >27°C vs <27°C  precipitation | Statistically significant:  RR=1.42£ (p<0.001)  *With higher PPR in football*  Statistically significant:  RR=1.49£ (p=0.03)  *With higher PPR in rock concerts*  Statistically significant:  RR=1.22£ (p=0.004)  *With higher PPR for heat index >27°*  Not statistically significant:  p>0.05 £† | No |
| Morimura, 2004, Japan | PPR (N/1,000) | Multivariable model:  access to venue;  maximum capacity of stadium | Statistically significant with R²=0.66:  PPR = 0.8903 + 0.9742*access - 0.00002079*capacity (p<0.0001)£  *With higher PPR for difficult access and decreasing capacity* | Yes |
| Selig, 2013, USA | PPR (N/10,000) | Multivariable model:  mean temperature;  practice day vs race day;  precipitation | Statistically significant:  RR=1.04, 95%CI [1.004;1.08] (p=0.03)  RR=0.47, 95%CI[0.24;0.90] (p=0.02)  *With higher PPR for increasing temperature and for race days*  Statistically not significant:  RR=0.39, 95%CI [0.15;1.01] (p=0.05) ¥ | No |
| Tan, 2014, Singapore | PPR (N/10,000) | Multivariable model:  age (per y)  sex: male vs female  competitive vs non-competitive  distance: 21km vs 10km | PPR = -6.4 - 0.0161*age – 0.567*male + 0.698*competitive + 1.133*21km  Statistically significant:  β = -0.0161 ± 0.008 (p<0.05)  *With higher PPR for decreasing age*  Statistically significant:  β = -0.567 ± 0.17 (p<0.001)  *With higher PPR for female*  Statistically significant:  β = 0.698 ± 0.202 (p<0.001)  *With higher PPR for competitive*  Statistically significant:  β = 1.133 ± 0.182 (p<0.001)  *With higher PPR for 21km* | Yes |
| Westrol, 2017, USA | PPR (N/1,000) | Multivariable model:  genre  festival vs no festival  heat index (≥32.2°C vs <32.2°C)  precipitation vs no precipitation  day of the week | PPR = genre + 0.223*festival + 0.125*heat index  (genre: see Table 6)  Statistically significant:  β by genre £ (see Table 6)  *With higher PPR for country (p=0.04), heavy metal (p=0.001), hip-hop/rap (p<0.001), alternative rock (p<0.001), modern rock (p<0.001)*  Statistically significant:  β = 0.223£ (p=0.001)  *With higher PPR for festival*  Statistically significant:  β = 0.125£ (p=0.023)  *With higher PPR for heat index ≥32.2°C*  Not statistically significant:  β = -0.025£† (p=0.649)  Not statistically significant:  β = 0.003£† (p=0.955) | Yes |
