# Supplementary material for: Prediction modelling studies for medical usage rates in mass gatherings: A systematic review
Source: PLoS One. 2020 Jun 23;15(6):e0234977. doi: 10.1371/journal.pone.0234977 (PMC7310685; doi:10.1371/journal.pone.0234977)
Supplement: S2 Table — £ No raw data/SD’s available (or specify), effect size and CI cannot be calculated; † Imprecision (lack of data). (DOCX) [file pone.0234977.s008.docx]

| **Author, year, Country** | **Outcome** | **Predictor** | **Effect size** | **Full model equation available?** |
| --- | --- | --- | --- | --- |
| Arbon, 2001, Australia | Total number of persons transported to hospital (TTOTNUM) | Multivariable model including:  known or predicted(*) number of patient presentations;  seated vs. mobile;  bounded vs. unbounded  (*)predicted with a separate model on total number of patient presentations | Statistically significant with R²=0.34:  TTOTNUM = 0 + 0.011*presentations – 1.0*seated + 1.5*bounded  *With higher expected* TTOTNUM *for mobile, bounded manifestations, and for increasing number of known or predicted patient presentations* | Yes |
| Arbon, 2018, Australia | Total number of patients transported | Regression tree including:  mobility, type of manifestation; duration; attendance; temperature; humidity; | Regression tree: see Appendix C in the paper by Arbon et al. (2018) | Yes (cfr. Regression trees) |
| Selig, 2013, USA | TTHR (N/10,000) | Multivariable model:  mean temperature;  precipitation;  practice day vs race day; | Statistically not significant (p>0.05) £† | No |
| Westrol, 2017, USA | TTHR (N/1,000) | Multivariable model:  alcohol/drugs intoxication vs medical/traumatic injuries  traumatic injuries vs alcohol/drugs intoxication or medical injuries  alternative rock  country  other music genres than alternative rock or country  music festivals vs no music festivals  heat index (≥32.2°C vs <32.2°C) | Statistically significant (p<0.001) £†  *With higher TTHR for alcohol/drugs intoxication*  Statistically significant (p=0.004) £†  *With lower TTHR for traumatic injuries*  Statistically significant (p=0.017) £†  *With higher TTHR for alternative rock*  Statistically significant (p=0.033) £†  *With higher TTHR for country*  Statistically not significant (p>0.05) £†  Statistically not significant (p>0.05) £†  Statistically significant (p=0.008) £†  *With higher TTHR for higher heat index* | No |
