# Supplementary material for: Prediction modelling studies for medical usage rates in mass gatherings: A systematic review
Source: PLoS One. 2020 Jun 23;15(6):e0234977. doi: 10.1371/journal.pone.0234977 (PMC7310685; doi:10.1371/journal.pone.0234977)
Supplement: S3 Table — OR: odds ratio; CI: Confidence Interval; BMI: Body Mass Index; £ No raw data/SD’s available (or specify), effect size and CI cannot be calculated; ¥ Imprecision (large variability of results); † Imprecision (lack of data). (DOCX) [file pone.0234977.s009.docx]

| **Author, year, Country** | **Outcome** | **Predictor** | **Effect size** | **Full prediction equation available?** |
| --- | --- | --- | --- | --- |
| Schwabe, 2014, South Africa | Medical complications (general) | Multivariable model:  year of race;  gender;  age group;  running experience category;  running pace category | Statistically significant:  2008 (incidence: 3.81, 95%CI[2.75;5.27] vs 2011 (incidence: 6.21, 95%CI [4.75;8.12]) (p=0.0201)  2009 (incidence: 3.21, 95%CI [2.28;4.53]) vs 2011 (incidence: 6.21, 95%CI [4.75;8.12]) (p=0.0019)  2010 (incidence: 3.56, 95%CI [2.52;5.03]) vs 2011 (incidence: 6.21, 95%CI [4.75;8.12]) (p=0.0096)  *With higher incidence medical complications for 2011 (colder environmental conditions)*  Statistically significant:  Women >50y vs women ≤50y (p<0.0001) £  *With higher incidence medical complications for women >50y*  Statistically not significant:  ≤1 medals (incidence: 5.89, 95%CI[4.93;7.05]) vs >2 medals (incidence: 4.26, 95%CI [3.43;5.28]) (p>0.05)  Statistically not significant:  <6min/km (incidence: 3.79, 95%CI[2.87;4.99] vs 6-7 min/km (incidence: 3.54, 95%CI [2.65;4.73] vs >7 min/km (incidence: 5.53, 95%CI [4.39;6.96])  (p>0.05) | No |
|  | Medical complications (specific): postural hypotension | Multivariable model:  year of race;  Gender;  Age group;  Running experience;  Running pace  Running pace | Statistically not significant:  2008 (incidence: 0.72, 95%CI[0.35;1.52] vs 2011 (incidence: 1.58, 95%CI [0.95;2.62]) (p>0.05)  2009 (incidence: 0.75, 95%CI [0.38;1.50]) vs 2011 (incidence: 1.58, 95%CI [0.95;2.62]) (p>0.05)  2010 (incidence: 1.26, 95%CI [0.72;2.22]) vs 2011 (incidence: 1.58, 95%CI [0.95;2.62]) (p=0.0096)  Statistically significant:  Female (incidence: 1.52, 95%CI[1.05;2.21] vs Male (incidence: 0.67, 95%CI[0.40;1.13]) (p=0.0394)  *With higher incidence postural hypotension for women*  Statistically not significant:  16-30y (incidence: 1.04, 95%CI[0.64;1.70] vs >50 (incidence: 1.66, 95%CI [0.87;3.19]) (p>0.05)  31-40 (incidence: 0.73, 95%CI [0.37;1.47]) vs >50 (incidence: 1.66, 95%CI [0.87;3.19]) (p>0.05)  41-50 (incidence: 1.18, 95%CI [0.61;2.26]) vs >50 (incidence: 1.66, 95%CI [0.87;3.19]) (p>0.05)  Statistically not significant:  ≤1 medals (incidence: 1.04, 95%CI[0.68;1.60] vs >2 medals (incidence: 1.09, 95%CI [0.71;1.68]) (p>0.05)  Statistically significant:  6-7 min/km (incidence: 0.38, 95%CI [0.16;0.92]) vs >7 min/km (incidence: 1.46, 95%CI [0.94;2.29]) (p=0.0139)  *With higher incidence postural hypotension for slow running pace*  Statistically not significant:  <6 min/km (incidence: 0.77, 95%CI[0.42;1.43] vs >7 min/km (incidence: 1.46, 95%CI [0.94;2.29]) (p>0.05) | No |
|  | Medical complications (specific): musculoskeletal complications | Multivariable model:  Year of race;  Gender;  Age group;  Running experience;  Running pace | Statistically not significant (p>0.05) £†  Statistically not significant (p>0.05) £†  Statistically not significant (p>0.05) £†  Statistically not significant (p>0.05) £†  Statistically not significant (p>0.05) £† | No |
| van Poppel, 2016, The Netherlands | Running injuries in the half marathon | Multivariable model (R^2^=4.5%):  Gender;  BMI;  Training frequency;  Type of terrain;  Running experience (5-10 years vs >10 years);  Type of training (always interval vs sometimes);  Running experience (<5 years vs >10 years); | Statistically not significant (p>0.05) £†  Statistically not significant (p>0.05) £†  Statistically not significant (p>0.05) £†  Statistically not significant (p>0.05) £†  Statistically not significant:  OR 1.14, 95%CI [0.64;2.01] (p=0.66)  Statistically significant:  OR 0.67, 95%CI [0.33;0.81] (p<0.01)  *With higher PPR for sometimes interval*  Statistically significant:  OR 1.87, 95%CI [1.13;3.11] (p=0.02)  *With higher PPR for <5 years or running experience* | No |
| Woodall, 2010, Australia | Injury status (injury vs. non-injury) | Multivariable model (adjusted for duration of the manifestation, size of the manifestation, environment, alcohol, mobility, age, time of day):  season: summer vs spring  season: autumn vs spring  season: winter vs spring  sex: male vs female  type of the manifestation: sporting manifestations vs other  type of the manifestation: carnival/fete vs other  type of the manifestation: concert/rave vs other | Not statistically significant:  OR=0.56, 95%CI [0.3-1.1] ¥† (p=0.080)*  Not statistically significant:  OR=0.74, 95%CI [0.4-1.3] ¥† (p=0.32)*  Statistically significant:  OR=1.81, 95%CI [1.03-3.2] (p=0.040)*  *With higher risk on injury vs non-injury in winter*  Statistically significant:  OR=3.01, 95%CI [2.1-4.3] (p<0.0001)*  *With higher risk on injury vs non-injury in men*  Statistically significant:  OR=4.91, 95%CI [1.9-12.4] (p<0.001)*  *With higher risk on injury in sporting manifestations*  Not statistically significant:  OR=1.23, 95%CI [0.5-3.1] ¥† (p=0.67)*  Not statistically significant:  OR=1.53, 95%CI [0.6-4.3] ¥† (p=0.40)* | No |
