# Supplementary material for: Prediction modelling studies for medical usage rates in mass gatherings: A systematic review
Source: PLoS One. 2020 Jun 23;15(6):e0234977. doi: 10.1371/journal.pone.0234977 (PMC7310685; doi:10.1371/journal.pone.0234977)
Supplement: S2 File — (DOCX) [file pone.0234977.s011.docx]

**MEDLINE (via the Pubmed Interface):**

1. ("Anniversaries and Special Events"[mesh] OR “recreation”[mesh] OR "mass event"[TIAB] OR “mass events”[TIAB] OR "mass gathering"[TIAB] OR crowd[TIAB] OR crowds[TIAB] OR "mass gatherings"[TIAB] OR “special event”[TIAB] OR “special events”[TIAB] OR "sport event"[TIAB] OR “sport events”[TIAB] OR “sporting event”[TIAB] OR “sporting events”[TIAB] OR marathon[TIAB] OR "music event"[TIAB] OR “music events”[TIAB] OR concert[TIAB] OR concerts[TIAB] OR rave[TIAB] OR raves[TIAB] OR festival[TIAB] OR festivals[TIAB] OR occasion[TIAB] OR occasions[TIAB] OR “social events”[TIAB] OR “social events”[TIAB] OR recreation[TIAB])

2. (“health planning”[Mesh] OR "emergency medicine"[Mesh] OR “health planning”[TIAB] OR “emergency care planning”[TIAB] OR "Emergency Medical Services"[Mesh] OR emergencies[TIAB] OR "emergency care"[TIAB] OR "First Aid"[Mesh] OR "first aid"[TIAB] OR "emergency medicine"[TIAB])

3. (“forecasting”[Mesh] OR “regression analysis”[Mesh] OR "models, theoretical"[Mesh] OR “Risk Assessment”[Mesh] OR “ROC curve”[Mesh] OR prediction[TIAB] OR “predictive model”[TIAB] OR predict[TIAB] OR “forecasting”[TIAB] OR “regression analysis”[TIAB] OR “statistical regression”[TIAB] OR modeling[TIAB] OR modelling[TIAB] OR model[TIAB] OR models[TIAB] OR validation[TIAB] OR validate[TIAB] OR prognostic[TIAB] OR risk[TIAB] OR AUC[TIAB])

4. 1-3 AND

**Embase (via Embase.com interface)**

1. (‘mass gathering’/exp OR ‘sporting event’/exp OR ‘marathon’/exp OR ‘recreation’/exp OR ‘mass event’:ab,ti OR ‘mass events’:ab,ti OR ‘mass gathering’:ab,ti OR crowd:ab,ti OR crowds:ab,ti OR ‘mass gatherings’:ab,ti OR ‘special event’:ab,ti OR ‘special events’:ab,ti OR ‘sport event’:ab,ti OR ‘sport events’:ab,ti OR ‘sporting event’:ab,ti OR ‘sporting events’:ab,ti OR marathon:ab,ti OR ‘music event’:ab,ti OR ’music events’:ab,ti OR concert:ab,ti OR concerts:ab,ti OR rave:ab,ti OR raves:ab,ti OR festival:ab,ti OR festivals:ab,ti OR occasion:ab,ti OR occasions:ab,ti OR ‘social events’:ab,ti OR ‘social events’:ab,ti OR ‘recreation’:ab,ti)

2. (‘health care planning’/exp OR ‘health care facility’/exp OR ‘emergency health service’/exp OR ‘First Aid’/exp OR ‘emergency care’/exp OR ‘emergency medicine’/exp OR ‘health planning’:ab,ti OR ‘emergency care planning’:ab,ti OR emergencies:ab,ti OR ‘emergency care’:ab,ti OR ‘first aid’:ab,ti OR ‘emergency medicine’:ab,ti)

3. (‘forecasting’/exp OR ‘prediction and forecasting’/exp OR forecasting:ab,ti OR prediction:ab,ti OR ‘predictive model’:ab,ti OR predict:ab,ti OR ‘regression analysis’:ab,ti OR ‘regression’/exp OR ‘statistical regression’/exp Or ‘statistical regression’:ab,ti OR modeling:ab,ti OR modelling:ab,ti OR ‘model’/exp OR model:ab,ti OR models:ab,ti OR ‘risk Assessment’/exp OR ‘risk assessment’:ab,ti OR ‘validation’/exp OR ‘validation’:ab,ti OR validate:ab,ti OR prognostic:ab,ti OR ‘risk’/exp OR risk:ab,ti OR 'receiver operating characteristic'/exp OR 'receiver operating characteristic':ab,ti OR ‘ROC curve’:ab,ti OR 'area under the curve'/exp OR AUC:ab,ti OR ‘area under the curve’:ab,ti)

4. 1-3 AND

**Cochrane library**

1. ([mh "Anniversaries and Special Events"] OR [mh “recreation”] OR "mass event":ti,ab,kw OR “mass events”:ti,ab,kw OR "mass gathering":ti,ab,kw OR crowd:ti,ab,kw OR crowds:ti,ab,kw OR "mass gatherings":ti,ab,kw OR “special event”:ti,ab,kw OR “special events”:ti,ab,kw OR "sport event":ti,ab,kw OR “sport events”:ti,ab,kw OR “sporting event”:ti,ab,kw OR “sporting events”:ti,ab,kw OR marathon:ti,ab,kw OR "music event":ti,ab,kw OR “music events”:ti,ab,kw OR concert:ti,ab,kw OR concerts:ti,ab,kw OR rave:ti,ab,kw OR raves:ti,ab,kw OR festival:ti,ab,kw OR festivals:ti,ab,kw OR occasion:ti,ab,kw OR occasions:ti,ab,kw OR “social events”:ti,ab,kw OR “social events”:ti,ab,kw OR recreation:ti,ab,kw)

2. ([mh “health planning”] OR [mh "Emergency Medical Services"] OR [mh "First Aid"] OR [mh "emergency medicine"] OR “health planning”:ti,ab,kw OR “emergency care planning”:ti,ab,kw OR emergencies:ti,ab,kw OR "emergency care":ti,ab,kw OR "first aid":ti,ab,kw OR "emergency medicine":ti,ab,kw)

3. ([mh “forecasting”] OR [mh “regression analysis”] OR [mh "models, theoretical"] OR [mh “Risk Assessment”] OR prediction:ti,ab,kw OR “predictive model” :ti,ab,kw OR predict:ti,ab,kw OR “forecasting”:ti,ab,kw OR “regression analysis” :ti,ab,kw OR “statistical regression”:ti,ab,kw OR modeling:ti,ab,kw OR modelling:ti,ab,kw OR model:ti,ab,kw OR models:ti,ab,kw OR validation:ti,ab,kw OR validate:ti,ab,kw OR prognostic:ti,ab,kw OR risk:ti,ab,kw OR “ROC curve”:ti,ab,kw OR AUC:ti,ab,kw)

4. 1-3 AND

**CINAHL**

1. (MH "Anniversaries and Special Events" OR MH “recreation” OR TI "mass event" OR TI “mass events” OR TI "mass gathering" OR TI crowd OR TI crowds OR TI "mass gatherings" OR TI “special event” OR TI “special events” OR TI "sport event" OR TI “sport events” OR TI “sporting event” OR TI “sporting events” OR TI marathon OR TI "music event" OR TI “music events” OR TI concert OR TI concerts OR TI rave OR TI raves OR TI festival OR TI festivals OR TI occasion OR TI occasions OR TI “social events” OR TI “social events” OR TI recreation OR AB "mass event" OR AB “mass events” OR AB "mass gathering" OR AB crowd OR AB crowds OR AB "mass gatherings" OR AB “special event” OR AB “special events” OR AB "sport event" OR AB “sport events” OR AB “sporting event” OR AB “sporting events” OR AB marathon OR AB "music event" OR AB “music events” OR AB concert OR AB concerts OR AB rave OR AB raves OR AB festival OR AB festivals OR AB occasion OR AB occasions OR AB “social events” OR AB “social events” OR AB recreation)

2. (MH “health planning” OR MH "Emergency Medical Services" OR MH "First Aid" OR MH "emergency medicine" OR TI “health planning” OR TI “emergency care planning” OR TI emergencies OR TI "emergency care" OR TI "first aid" OR TI "emergency medicine" OR AB “health planning” OR AB “emergency care planning” OR AB emergencies OR AB "emergency care" OR AB "first aid" OR AB "emergency medicine")

3. (MH “forecasting” OR MH “regression analysis” OR MH "models, theoretical" OR MH “Risk Assessment” OR MH “ROC curve” OR TI prediction OR TI “predictive model” OR TI predict OR TI “forecasting” OR TI “regression analysis” OR TI “statistical regression” OR TI modeling OR TI modelling OR TI model OR TI models OR AB prediction OR AB “predictive model” OR AB predict OR AB “forecasting” OR AB “regression analysis” OR AB “statistical regression” OR AB modeling OR AB modelling OR AB model OR AB models OR TI validation OR TI validate OR TI prognostic OR TI risk OR TI AUC OR AB validation OR AB validate OR AB prognostic OR AB risk curve” OR AB AUC)

4. 1-3 AND

**Web of Science**

1. TS=("mass event" OR “mass events” OR "mass gathering" OR crowd OR "mass gatherings" OR “special event” OR “special events” OR "sport event" OR “sport events” OR “sporting event” OR “sporting events” OR marathon OR "music event" OR “music events” OR concert OR rave OR festival OR occasion OR “social events” OR “social events” OR “recreation” OR recreation)

2. TS=(“health planning” OR “health care planning” Or “health care facility” Or “emergency health service” OR “emergency care planning” OR "Emergency Medical Services" OR emergencies OR "First Aid”)

3. TS=(“forecasting” OR prediction OR “predictive model” OR predict OR “regression analysis” OR “statistical regression” OR modeling OR “Risk Assessment” OR validation OR validate OR prognostic OR risk OR “ROC curve” OR AUC)

4. 1-3 AND

**Scopus**

1. (INDEXTERMS("mass gathering") OR INDEXTERMS("Anniversaries and Special Events") OR INDEXTERMS("sporting event") OR INDEXTERMS("marathon") OR INDEXTERMS("recreation") OR TITLE-ABS-KEY("mass event") OR TITLE-ABS-KEY("mass events") OR TITLE-ABS-KEY("mass gathering") OR TITLE-ABS-KEY(crowd) OR TITLE-ABS-KEY(crowds) OR TITLE-ABS-KEY("mass gatherings") OR TITLE-ABS-KEY("special event") OR TITLE-ABS-KEY("special events") OR TITLE-ABS-KEY("sport event") OR TITLE-ABS-KEY("sport events") OR TITLE-ABS-KEY("sporting event") OR TITLE-ABS-KEY("sporting events") OR TITLE-ABS-KEY(marathon) OR TITLE-ABS-KEY("music event") OR TITLE-ABS-KEY("music events") OR TITLE-ABS-KEY(concert) OR TITLE-ABS-KEY(concerts) OR TITLE-ABS-KEY(rave) OR TITLE-ABS-KEY(raves) OR TITLE-ABS-KEY(festival) OR TITLE-ABS-KEY(festivals) OR TITLE-ABS-KEY(occasion) OR TITLE-ABS-KEY(occasions) OR TITLE-ABS-KEY("social events") OR TITLE-ABS-KEY("social events") OR TITLE-ABS-KEY("recreation")

2. (INDEXTERMS("health planning") OR INDEXTERMS("health care planning") OR INDEXTERMS("health care facility") OR INDEXTERMS("emergency health service") OR INDEXTERMS("Emergency Medical Services") OR INDEXTERMS("emergency care") OR INDEXTERMS("First Aid") OR INDEXTERMS("emergency medicine") OR TITLE-ABS-KEY("health planning") OR TITLE-ABS-KEY("emergency care planning") OR TITLE-ABS-KEY("emergencies") OR TITLE-ABS-KEY("emergency care") OR TITLE-ABS-KEY("first aid") OR TITLE-ABS-KEY("emergency medicine")

3. (INDEXTERMS("forecasting") OR INDEXTERMS("prediction and forecasting") OR INDEXTERMS("regression") OR INDEXTERMS("regression analysis") OR INDEXTERMS("statistical regression") OR INDEXTERMS("models, theoretical") OR INDEXTERMS("model") OR INDEXTERMS("Risk Assessment") OR INDEXTERMS("ROC curve") OR INDEXTERMS("validation") OR INDEXTERMS("risk") OR INDEXTERMS("receiver operating characteristic") OR INDEXTERMS("area under the curve") OR TITLE-ABS-KEY(prediction) OR TITLE-ABS-KEY("predictive model") OR TITLE-ABS-KEY(predict) OR TITLE-ABS-KEY("forecasting") OR TITLE-ABS-KEY("regression analysis") OR TITLE-ABS-KEY("statistical regression") OR TITLE-ABS-KEY(modeling) OR TITLE-ABS-KEY(modelling) OR TITLE-ABS-KEY(model) OR TITLE-ABS-KEY(models) OR TITLE-ABS-KEY(validation) OR TITLE-ABS-KEY(validate) OR TITLE-ABS-KEY(prognostic) OR TITLE-ABS-KEY(risk) OR TITLE-ABS-KEY(AUC) OR TITLE-ABS-KEY("risk assessment") OR TITLE-ABS-KEY("receiver operating characteristic") OR TITLE-ABS-KEY("area under the curve"))

4. filter: exclude conference paper, conference review, book (chapter), editorial, note, letter

5. 1-3 AND
