# Supplementary material for: Prediction modelling studies for medical usage rates in mass gatherings: A systematic review
Source: PLoS One. 2020 Jun 23;15(6):e0234977. doi: 10.1371/journal.pone.0234977 (PMC7310685; doi:10.1371/journal.pone.0234977)
Supplement: Attachment — Submitted filename: REVIEWERS COMMENTS.pdf [file pone.0234977.s012.pdf]

# REVIEWER'S COMMENTS

## Comments to the editor:

Thank you so much for giving me the opportunity to review this important manuscript.

This work provides information to aid effective planning of mass gatherings, which are on the increase in every society. The authors pointing to the fact the United Nations, through the World Health Organization, is paying serious attention to mass gatherings, with the recommendation of mass gathering medicine for all the WHO member states. For this reason, I score this manuscript high and suggest that it should be accepted for publication, subject to addressing the obvious gaps I highlighted in each of the sections below (introduction, results, discussion and conclusion):

## Introduction Section

The justification for the study is weak. The main justification that the authors provide for this study is contained in the two sentences below:

***"Since mass gatherings attended by large crowds have become a more frequent feature of society, mass gathering medicine was highlighted as a new discipline at the World Health Assembly of Ministers of Health in Geneva in May 2014. As a consequence, the amount of international initiatives and meetings on mass gathering medicine has increased over the past decade as has the number of experts and the amount of publications on pre-event planning and surveillance for mass gathering".***

However, the frequency of mass gathering, the recommendation of mass gathering medicine for the WHO member states by the WHO and the increase in international initiatives and meetings on mass gathering medicine, are not a strong public health/clinical problem to justify a study. It is important to clearly state the specific clinical, public health and economic risks associated with mass gatherings to justify this study. For instance, do mass gatherings increase the risks of diseases and mortality to the participants and the larger society? What are the economic and social problems associated with mass gatherings that may dove tail into major health problems? The authors have mentioned "Patient presentation rate (PPR), Transfer to Hospital Rate (TTHR) and new injuries" as the major health problems but these alone cannot attract the attention of policy makers to invest in interventions to minimize the risks of mass gatherings. In the developing countries, there are far more important public health and developmental problems than PPR, TTHR and new injuries, to attract the attention of government. Therefore the authors must show what the society is suffering on account of mass gathering to strongly justify this study.

The authors also need to show how poor planning of events increase the risks associated with mass gathering. This is so because the findings of the study are intended to promote effective pre-event planning. If policy makers cannot see the risks of poor event planning, why should they care to make policies that would encourage effective planning?

**Results Section**

The result section has six sections: (1) Study selection, (2) factors that predict patients' presentation (rate) (3) Factors that predict transfer to hospital (rate) (4) factors that predict the incidence of new sport injuries (5) external model validation studies and (6) Graded assessment. My comments on each of the sections are provided below:

**(1) Study selection,**

This section is too detailed and elaborate. Some of the information provided in this section should be taken to the methods section. For instance, the following sentences can go to the methods:

**"Fig 1** represents the study selection process used" – This sentence has nothing to do with results

"A mix of different types of mass events was included such as sports (spectator) events (e.g. soccer games, auto races, (half-) marathon), music concerts (indoor/outdoor), fete/carnivals, public exhibitions and ceremonial events". This sentence has nothing to do with results. It's a method statement.

"Data were collected in 8 studies between 2005-2015, in 7 studies between 1995-2005, and in 2 studies between 1980-1995".

The above sentences are not part of the results and therefore should not be included in the results.

We can summarize the entire section by providing only significant information that tally with the aim of the study. Here is my recommendation below:

**"We included 17 cross sectional studies and more than 1,700 mass events (more than 48 million people attending these vents). Majority of the studies (n=13, 76%) were conducted in the USA (n=9, 52.94%) and Australia (n=4, 23.52%); with a few studies from Japan (n=1, 5.88%), Singapore (n=1, 5.88%), South Africa (n=1, 5.88%) and The Netherlands (n=1, 5.88%). Most of the studies [n= 15, 88.23%) measured influx at first aid posts as an outcome, while nearly half of the studies (n=7, 41.17%) focused on transfer to the hospital, with a few studies (n=3, 17.64%) assessing the incidence of new (non-)medical injuries/complications as the outcome. Almost all the studies investigated whether at least one of the following environmental candidate predictors were associated with medical usage (rates): Thirteen studies (76.47%) assessed weather; 12 (70.58%) studies assessed crowd size; etc"**

**(2) Factors that predict patients' presentation (rate)**

Again, there are several sentences in this section that should be moved to the discussion section. Any sentence that clarifies or justifies an observation should go to the discussion, e. g., this sentence:

"In a recent study by Arbon et al., temperature (i.e. <23.5°C vs ≥23.5°C and <25.5°C vs ≥25.5°C) was included in a non-linear regression tree model with a lower total number of patient presentations in case of higher temperatures". This sentence is not a result but supporting information. Please note that in the result section, the guidelines requires only the findings to be presented without justifying or supporting them with references. We can do this in the discussion section to illustrate the consistency of our findings with the findings of other researchers.

In order to portray the significance of the factors and their contributions, I suggest that percentages (%) should be used instead of just mentioning the factors and the number of studies that reported them. If the data will allow, the P-values should also be reported in all the predictable factors. Importantly, note that this sections deals with only the predictors and not the studies.

**(3) Factors that predict transfer to hospital (rate)**

The comments made in (2) above applies here also.

**(4) Factors that predict the incidence of new sport injuries**

The comments made in (2) above applies here also.

## Discussion

The first paragraph of the discussion is a repetition of information that is well documented in previous sections. Perhaps, starting the discussion with the major findings of this study will be more exciting as an introduction to the discussion than repeating the work that was done.

The findings of the study should also be discussed in line with the findings of other authors, since it was reported in the introduction that there are has been an "increased over the past decade in the number of experts and the amount of publications on pre-event planning and surveillance for mass gathering".

## Conclusion

I suggest that the conclusion should be revised to show that the two major aims of conducting the study have been achieved. The implications of the findings should also be stated.
