# Supplementary material for: Prediction modelling studies for medical usage rates in mass gatherings: A systematic review
Source: PLoS One. 2020 Jun 23;15(6):e0234977. doi: 10.1371/journal.pone.0234977 (PMC7310685; doi:10.1371/journal.pone.0234977)
Supplement: Attachment — Submitted filename: 20200604_Comments Reviewers_point-by-point answers.docx [file pone.0234977.s014.docx]

Additional Editor Comments

This is an interesting manuscript. However, before beinig considered for publication some minor issues in the methods section should be clarified.

1. Please clarify how many reviewers performed the risk of bias assessment. In addition, the risk of bias assessment should be described in an own section. In general, please try to use headings as suggested in PRISMA.

*Answer:*

We revised the manuscript and 1) added that the 2 reviewers independently performed the risk of bias assessment (page 8, lines 26-27), 2) restructured the information of the methods and results section into paragraphs that are now ordered according to the PRISMA checklist (including a separate paragraph on risk of bias assessment).

2. GRADE was not developed to assess studies on risk prediction models. Therefore, the conduct of the GRADE assessment should be described in much more detail.

*Answer:*

We agree with the Editor and further elaborated on using the GRADE approach to rate the certainty of the evidence in prediction modelling studies (see methods section, pages 10-11).

Reviewer #1:

The authors have addressed some of my original comments, however a few points remain.

1. Page 5, lines 7-9 (In relation to my original comment 1): The ‘external validation’ studies (see later comment) still do not fit with the current aims. Perhaps break down the objectives, for example:

1) To identify multivariable prediction models for any outcome at mass gatherings.

2) To summarise evidence for individual predictors of outcomes at mass gatherings.

3) To summarise predictive performance of the models in the development studies as well as when applied to new settings.

Measures like R2 would then be for objective 3 and you may wish to summarise performance of models under a separate heading in the results.

*Answer:*

We agree with the reviewer and rephrased the study objectives at the end of the introduction (Page 5, lines 7-11). In the results section, we rephrased the subheading ‘external model validation studies’ into ‘predictive performance of the models’ and added relevant information (e.g. R2) from the prediction development studies here. (Page 21, lines 12-22).

2. Page 6, line 29 & Page 8, line 17: While I appreciate that the authors are considering sample size as part of their risk of bias assessment for studies, I’m still uneasy with the authors saying that they only extracted information for ‘statistically significant’ predictors. If meta-analyses were possible, you wouldn’t exclude non-significant effects as the aim is to pool all the evidence for the predictor so why exclude them here, just because a meta-analysis is not possible in the end? Predictors may appear significant by chance in some studies (overfitting) and not in other studies (when they are important) due to small sample size or because it is highly correlated with another variable in the model. In fact, I think the authors have already addressed this based on another reviewer's comment and now report for all predictors so they could just remove mention of significance in the methods.

*Answer:*

Correct, we changed this information (i.e. both the statistically significant and non-statistically significant factors from the multivariate models are relevant/important) throughout the manuscript, including at page 6, lines 17-18, and at page 9, line 1.

3. External validation studies: I don’t think the authors understood my original comment 8. Because the observations (data points) here are mass gatherings, seeing how well the model predicted for a single mass gathering is just a prediction to a new observation, rather than an external validation as such. It’s the same as predicting risk of death for a single person and comparing to if they died or not – the uncertainty is huge and you would need to predict for many individuals to say whether the model predicts well or not. So here, you would need a dataset with many mass gatherings, similar to what was used to develop the model. The model could over and under predict for individual gatherings but calibrate well overall, so we just don’t know from one or even 3 gatherings. Therefore, I suggest you refer to these predictions as something other than external validation. Maybe refer to it as ‘application of prediction models for mass gatherings’ or something similar and discuss that not much can be concluded from individual predictions but that the models would need to be validated in larger datasets of mass gatherings.

*Answer:* We thank the reviewer for this clarification and full agree with this comment. Therefore, we rephrased/removed (where needed) the term validation throughout the manuscript when reporting/discussing on these 3 studies, including that 1) no validation studies of prediction models in big data sets of mass gatherings were identified, 2) applications of prediction models of Arbon/Zeitz in few mass gatherings were present in 3 studies and 3) future validation studies in big data sets is needed.
